# Brain function in iNOS knock out or iNOS inhibited (l-NIL) mice under endotoxic shock

**DOI:** 10.1186/s40635-014-0024-z

**Published:** 2014-09-20

**Authors:** Hanna Schweighöfer, Christoph Rummel, Konstantin Mayer, Bernhard Rosengarten

**Affiliations:** Department of Neurology, Justus-Liebig-University Giessen, Klinikstrasse 33, 35392 Giessen, Germany; Department of Veterinary Physiology and Biochemistry, Justus-Liebig-University Giessen, Frankfurter Strasse 100, 35392 Giessen, Germany; Department of Internal Medicine II, Justus-Liebig-University Giessen, Klinikstrasse 33, 35392 Giessen, Germany

**Keywords:** Inflammation, Somatosensory-evoked potentials, Neurovascular coupling, Nitric oxide synthase, iNOS knock out

## Abstract

**Background:**

Microcirculatory dysfunction due to excessive nitric oxide production by the inducible nitric oxide synthase (iNOS) is often seen as a motor of sepsis-related organ dysfunction. Thus, blocking iNOS may improve organ function. Here, we investigated neuronal functional integrity in iNOS knock out (−/−) or l-NIL-treated wild-type (wt) animals in an endotoxic shock model.

**Methods:**

Four groups of each 10 male mice (28 to 32 g) were studied: wt, wt + lipopolysaccharide (LPS) (5 mg/kg body weight i.v.), iNOS(−/−) + LPS, wt + LPS + l-NIL (5 mg/kg body weight i.p. 30 min before LPS). Electric forepaw stimulation was performed before LPS/vehicle and then at fixed time points repeatedly up to 4.5 h. N1-P1 potential amplitudes as well as P1 latencies were calculated from EEG recordings. Additionally, cerebral blood flow was registered using laser Doppler. Blood gas parameters, mean arterial blood pressure, and glucose and lactate levels were obtained at the beginning and the end of experiments. Moreover, plasma IL-6, IL-10, CXCL-5, ICAM-1, neuron-specific enolase (NSE), and nitrate/nitrite levels were determined.

**Results:**

Decline in blood pressure, occurrence of cerebral hyperemia, acidosis, and increase in lactate levels were prevented in both iNOS-blocked groups. SEP amplitudes and NSE levels remained in the range of controls. Effects were related to a blocked nitrate/nitrite level increase whereas IL-6, ICAM-1, and IL-10 were similarly induced in all sepsis groups. Only CXCL-5 induction was lower in both iNOS-blocked groups.

**Conclusions:**

Despite similar hyper-inflammatory responses, iNOS inhibition strategies appeared neurofunctionally protective possibly by stabilizing macro- as well as microcirculation. Overall, our data support modern sepsis guidelines recommending early prevention of microcirculatory failure.

## Background

Sepsis and systemic inflammatory response syndromes (SIRS) are the leading causes of mortality in intensive care units [1,2]. Excessive production of nitric oxide (NO) by the inducible nitric oxide synthase (iNOS) plays a crucial role in early inflammatory syndromes [3-5].

In the brain, NO triggers several temporally cascaded negative effects. Within minutes to hours, microvascular dysfunction occurs resulting in an inappropriate blood supply of neurons [6-8]. As a consequence, levels of hypoxia-induced factor (HIF)-2 alpha increase, somatosensory-evoked potential amplitudes decline, and neuronal (neuron-specific enolase, NSE) and astrocytic (S100B) destruction markers increase 4 h after an endotoxin challenge [9]. After about 6 to 8 h, NO starts to affect mitochondrial function leading to an impaired aerobic glycolysis with energy depletion in neurons [10]. Moreover, NO is also involved in delayed neuronal apoptosis occurring 24 to 48 h following the insult [11]. Systemically, excessive NO levels lead to hypotension [12,13], microcirculatory dysfunction [14], and refractoriness to vasopressor catecholamines [15].

Previously, animals treated with selective iNOS inhibitors or transgenic mice deficient in iNOS had less hypotension and preserved microvascular reactivity under septic conditions [16,17]. Furthermore, iNOS inhibition stabilized also the brain circulation: The neurovascular coupling was stabilized during an endotoxin challenge using 1,400 W as a selective iNOS inhibitor [7]. The neurovascular coupling denotes a brain intrinsic regulative principle, which adapts the local cerebral blood flow in accordance with the metabolic needs (i.e. activity) of underlying neurons [18]. However, results did not clearly favor a 1,400-W therapy since 1,400 W had direct negative effects on somatosensory-evoked potential (SEP) amplitudes [7]. Interestingly, the effect was only seen under LPS challenge but not under control conditions. Thus, the question arises whether the negative effect on SEP was simply an adverse effect of the substance 1,400 W itself, or if it was related to the iNOS inhibition in general. To further address this issue, we studied the effects of endotoxic shock on SEP in iNOS knock out(−/−) or l-NIL inhibited mice.

## Methods

### General preparation

All procedures performed on the animals were in strict accordance with the National Institutes of Health Guide for Care and Use of Laboratory Animals and approved by the local Animal Care and Use Committee.

Experiments were carried out with wild-type (wt) C57BL6N or iNOS(−/−) C57BL6J adult male mice (28 to 32 g), as given below in detail. In separate experiments with five mice in each group, we tested effects of l-NIL in wt mice and stability of recordings in iNOS(−/−) mice, and studied inflammatory and neurophysiological responses to the LPS challenge in C57BL6J mice.

Mice were initially anesthetized with 1.5% to 3% isoflurane in a 7:3 N_2_O/O_2_ mixture of gases, tracheotomized, paralyzed with pancuronium bromide (0.2 mg/kg/h), and artificially ventilated (Minivent, Harvard Apparatus, South Natick, MA, USA). Arterial blood gas analyses and pH were measured at the beginning and the end of experiments (blood gas analyzer model Rapidlab 348, Bayer Vital GmbH, Fernwald, Germany) together with glucose and lactate levels (Glukometer Elite XL, Bayer Vital GmbH, Fernwald, Germany; Lactate pro, Arkray Inc. European Office, Düsseldorf, Germany). Glucose was kept in the physiological range by injections of 0.1 ml 20% glucose i.p. as needed. The right femoral artery and vein were cannulated for blood pressure recording, blood sampling, and drug administration. Rectal body temperature was maintained at 37°C using a feedback-controlled heating pad (Haake, Karlsruhe, Germany).

The head of the animals was fixed in a stereotaxic frame. After a median incision, the bone over the left parietal cortex was exposed allowing EEG and transcranial laser-Doppler flow (LDF) recording. Electric brain activity was recorded monopolarily with an active AgCl-electrode over the somatosensory forepaw area and an indifferent AgCl-electrode placed at the nasal bone [19]. Signals were recorded and amplified (BPA Module 675, HSE, March-Hugstetten, Germany) and SEP was averaged using the Neurodyn acquisition software (HSE, March-Hugstetten, Germany). The LDF probe (BRL-100, Harvard Apparatus, MA, USA) was placed laterally to the cortical electrode.

Approximately 60 min before the stimulation experiments, isoflurane/N_2_O anesthesia was discontinued and replaced by intravenous application of α-chloralose (60 mg/kg bw i.v. bolus) (Sigma-Aldrich Chemie GmbH, Taufkirchen, Germany). Anesthesia was continued by continuously administrating chloralose intravenously (30 mg/kg/h). During experiments, the animals were ventilated with nitrogen/oxygen mixture of 1/1.

### Neurophysiological measurements

Somatosensory stimulation was carried out with electrical pulses applied using small needle electrodes inserted under the skin of the right forepaw (PSM Module 676, HSE, March-Hugstetten, Germany). The right forepaw was electrically stimulated with rectangular pulses of 0.3 ms width and a repetition frequency of 2 Hz for 30 s. The stimulation current was kept constant at 1.5 mA to prevent systemic blood pressure changes [6,7]. From the averaged typical SEP responses, we calculated the N1-P1 amplitude differences and P1 latencies for further statistical comparisons.

### Clinical chemistry

At the end of the experiments, blood samples were collected into tubes containing heparin (Ratiopharm GmbH, Ulm, Germany) and immediately centrifuged, and plasma was stored at −80°C until analyses. The NSE levels were determined using an enzyme-linked immunosorbent assay (NSE EIA kit; Hoffmann-La Roche, Basel, Switzerland). Cytokine analysis was performed for IL-6 and IL-10 using commercial rat ELISA kits (BD Bioscience, Heidelberg, Germany). In addition, CXCL-5, a chemotactic chemokine, and ICAM-1, an endothelial activation marker, were determined according to the recommendation of the manufacturer (R&D Systems, Wiesbaden, Germany).

NO metabolite (nitrite and nitrate) concentrations were determined using NOA Sievers 280 (FMI GmbH, Seeheim, Germany) according to the manufacturer's instructions. Briefly, NO reaction products in plasma samples were reduced by vanadium chloride. Resulting gaseous NO was detected by NOA Sievers 280, which was connected to a computer for data transfer and analysis by NOAWIN32 software (DeMeTec, Langgöns, Germany).

### Study design

Each mouse (10 per group) was subjected to one of the following groups: wt control, wt + 5 mg/kg LPS (lipopolysaccharide from *Escherichia coli*, O111:B4, Sigma-Aldrich Chemie GmbH, Germany), wt + l-NIL + LPS, iNOS(−/−) + LPS. LPS was dissolved in 0.1 ml 0.9% NaCl and injected/infused within 2 to 3 min. The control group received 0.1 ml vehicle. A moderate volume therapy of 0.1 to 0.6 ml/kg/h 0.9% NaCl was allowed in all groups. In the l-NIL group, l-NIL was injected after neurophysiological baseline recording and 30 min before sepsis induction at a dose of 5 mg/kg body weight i.p.

SEPs, LDF signal, and blood pressure were measured up to 270 min before and after LPS application. This limited time window was chosen according to previous studies; it was shown that the cerebral autoregulation stays intact and that blood pressure levels remain above the lower limit of the cerebral autoregulative range for this whole time period [20,21].

### Statistics

If appropriate, a two-way ANOVA was performed to assess differences within and between groups. In case of significance, a Fisher *post hoc* test was applied. If assumptions of normal distribution and equality of variances could not be assured, a nonparametric Friedman test was undertaken instead (Statview, SAS, Cary, NC, USA). The significance level was set to *p* < 0.05.

The sample size was calculated with G-Power 3.1.3 (Faul, University of Kiel, Kiel, Germany). Assuming an effect size of 0.7 from previous reports, a total sample size of 40 animals was calculated to determine a significant difference between SEP amplitudes with an alpha error of 0.05 and a power of 0.95 between the four groups.

## Results

### General results

Hemodynamic and neurophysiological parameters were stable in wt + l-NIL as well as in iNOS(−/−) mice over the entire study window of 4.5 h. Responses to LPS did not differ between C57BL6N or C57BL6J mice (data not shown), and no mouse died from the slow LPS injection.

Table 1 shows the group averaged data for pO_2_, pCO_2_, pH, glucose, lactate, and hematocrit. Compared to control conditions, significant changes occurred in lactate and pH levels in the LPS groups. In the LPS groups, lactate levels increased to values in the range between 3.2 and 3.8 mmol/l but no significant differences were observed between the LPS groups. pH levels typically decreased in all LPS groups. However, values reached only significance in the wt + LPS group, whereas the iNOS-blocked groups showed only a trend to lower levels.

**Table 1:** **Gro** **up-averaged data for glucose, lactate, pH, pO2, pCO2, and hematocrit for all groups**

	**Glucose**	**Lactate**	**pH**	**pO**_**2**_	**pCO**_**2**_	**Hematocrit**
	**(mg/dl)**	**(mmol/l)**		**(mmHg)**	**(mmHg)**	**(%)**
Control	84 ± 12	1.5 ± 1	7.4 ± 0.03	170 ± 12	34 ± 6	46 ± 4
Wt + LPS	83 ± 14	3.2 ± 2*	7.2 ± 0.2****	180 ± 20	35 ± 8	45 ± 6
Wt + LPS + l-NIL	90 ± 17	3.2 ± 2*	7.3 ± 0.1	190 ± 25	36 ± 9	49 ± 6
iNOS(−/−) + LPS	78 ± 8	3.8 ± 2*	7.3 ± 0.1	177 ± 19	33 ± 5	47 ± 4
ANOVA	ns	*p* < 0.05	*p* < 0.0005	ns	ns	ns

Data are given as mean ± standard deviation (SD) together with statistical results. In case of a significant ANOVA, the *post hoc* statistical test results to baseline are given as *p < 0.05, ***p < 0.005, ****p < 0.0001. No significant (ns) differences were seen between sepsis groups.

Data from the pro-inflammatory cytokine IL-6, the anti-inflammatory cytokine IL-10, the chemokine CXCL-5, and the endothelial activation marker ICAM are shown together with the neuronal cell destruction marker (NSE) as well as nitrate/nitrite levels (NO) in Table 2. IL-6, IL-10, and ICAM levels increased significantly without differences between LPS groups. CXCL-5 was also significantly induced in all LPS groups. However, iNOS blocking lowered CXCL-5 levels in both groups by nearly 50%. However, NSE levels did not differ between control group and LPS groups. Nitrate/nitrite levels significantly increased in the wt + LPS group (330 ± 130 mmol/ml vs. control, 120 ± 50 mmol/ml; *p* < 0.001), whereas they did not differ from control in the l-NIL or iNOS(−/−) LPS groups.

**Table 2:** **Cytok** **ine, chemokine, and endothelial activation markers together with the neuronal destruction marker**

	**NSE**	**IL-6**	**IL-10**	**ICAM**	**CXCL-5**	**Nitrate/nitrite**
	**(ng/ml)**	**(ng/ml)**	**(ng/ml)**	**(ng/ml)**	**(ng/ml)**	**(μmol/l)**
Control	10 ± 3	1 ± 0.6	0.2 ± 0.1	84 ± 15	0.3 ± 0.3	120 ± 50
Wt + LPS	14 ± 4 (*p* = 0.08)	224 ± 81****	3.6 ± 1.5****	156 ± 20****	12 ± 6****	330 ± 130***
Wt + LPS + l-NIL	13 ± 2	175 ± 82****	1.8 ± 0.7*^, ##^	141 ± 12****	7 ± 2****^, ##^	150 ± 33
iNOS(−/−) + LPS	14 ± 3	243 ± 32****	2.6 ± 1.5**	145 ± 11****	6 ± 1****^, ##^	41 ± 10
ANOVA	ns	*p* < 0.0001	*p* < 0.0005	*p* < 0.0001	*p* < 0.0001	*p* < 0.0001

Cytokine, chemokine, and endothelial activation markers together with the neuronal destruction marker are given as mean ± SD together with statistical results. In case of a significant ANOVA, the *post hoc* statistical test results to baseline are given as **p < 0.01, ***p < 0.001, ****p < 0.0001. Statistical significant differences to wt + LPS in the LPS groups are given as ^##^ *p* < 0.01. ns, not significant.

### Neurofunctional results

**Figure 1:**
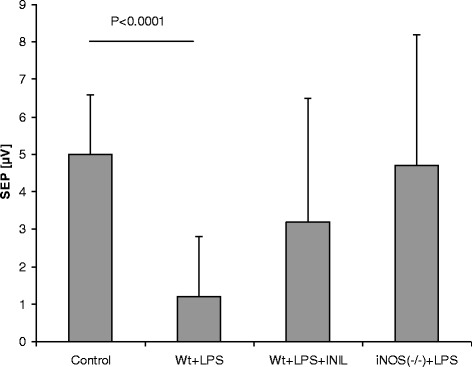
**Group-averaged data of SEP amplitudes, given as mean ± SD together with statistical results.** Whereas l-NIL and iNOS(−/−) groups presented stable responses, SEP significantly declined in the untreated LPS group. Data show a neurofunctionally protective effect of specific iNOS inhibition.

**Figure 2:**
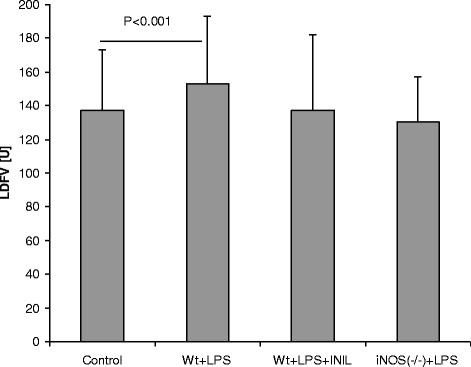
**Group-averaged data for LDFV responses, given as mean ± SD together with statistical results.** Whereas l-NIL and iNOS(−/−) groups presented stable Laser-Doppler flow velocity (LDFV) levels, significant hyperemia occurred in the untreated LPS group.

Table 3 contains the group data for blood pressure together with the resting LDF signal, N2-P1 potential amplitudes, and P1 latency. Mean blood pressure decreased significantly in the wt + LPS group (56 ± 21 mmHg vs. control, 73 ± 17 mmHg; *p* < 0.05), whereas it remained stable in the l-NIL and iNOS(−/−) LPS groups. Moreover, the occurrence of cerebral hyperemia was prevented. In the wt + LPS group, the resting cerebral blood flow increased by nearly 30% (control, 137 ± 36 U vs. wt + LPS, 180 ± 40 U; *p* < 0.0001). N2-P1 amplitudes declined significantly in the wt + LPS group (1.2 ± 1.6 μV vs. control 5 ± 1.6 μV; *p* < 0.0001), whereas no significant adverse effect was seen in the other LPS groups (Figure 1). P1 latencies did not differ between groups. Figure 2 indicates the results of the resting flow velocity levels in the brain for the different groups. Occurrence of LPS-related cerebral hyperemia was prevented by iNOS inhibitory strategies.

**Table 3:** **Gr** **oup-averaged data for mean BP, SEP, P1 latencies, and resting LDFV signal**

	**Mean BP (mmHg)**		**SEP (μV)**		**P1 latency (ms)**		**LDFV (U)**	
	**Base**	**End**	**Base**	**End**	**Base**	**End**	**Base**	**End (change to baseline)**
Control	85 ± 5	73 ± 17	6.6 ± 2.3	5 ± 1.6	9.4 ± 0.5	9.1 ± 0.8	144 ± 30	137 ± 36 (−5%)
Wt + LPS	90 ± 10	56 ± 21*	6.6 ± 3.3	1.2 ± 1.6****	9.3 ± 0.7	9.4 ± 0.1	153 ± 34	180 ± 40*** (+18%)
Wt + LPS + l-NIL	86 ± 8	68 ± 20	6.8 ± 2.2	3.2 ± 3.3	9.4 ± 0.7	9.3 ± 0.1	151 ± 32	137 ± 45 (−10%)
iNOS(−/−) + LPS	92 ± 15	60 ± 21	7.7 ± 2.2	4.7 ± 3.5	9.5 ± 0.6	9.1 ± 0.9	157 ± 30	130 ± 27 (−17%)
ANOVA	ns	*p* < 0.05	ns	*p* < 0.001	ns	ns	ns	*p* < 0.05

Changes to baseline are also given for the LDFV at the end of experiments. Data are given as mean ± SD together with statistical results. In case of a significant ANOVA, the *post hoc* statistical test results to baseline are given as *p < 0.05, ***p < 0.005, ****p < 0.0001. No significant differences were seen between sepsis groups.

## Discussion

This is the first report showing a stabilization of neuronal functioning due to selected iNOS inhibition under an endotoxin challenge: corroborated by iNOS(−/−) experiments, l-NIL stabilized SEP (N2-P1) amplitudes during the first hours of LPS-mediated shock. Previously reported negative effects of 1,400 W on SEP are, therefore, most likely due to a substance/drug-specific effect.

We assume that the stabilization of the macro- as well as microcirculation might best explain the stabilizing effect on SEP amplitudes. Occurrence of cerebral hyperemia and a progressive decline in blood pressure were effectively blocked in the l-NIL and iNOS(−/−) group as nitrate/nitrite levels remained in the range of controls. Cerebral hyperemia is caused by an excessive iNOS-related NO production [22]. NO interferes with the neurovascular coupling, resulting in an unselected widening of resistance vessels leading to an uncontrolled perfusion of the capillary territory and at least to an inappropriate blood supply of active neurons [6,9,23]. Neurons react very sensitively towards an inadequate perfusion due to their high-energy demand and strong aerobic metabolism [3]. A mismatch of about 10% to 20% leads to neuronal dysfunction and protein synthesis disturbances in neurons if it lasts for minutes to hours [24]. Similarly, the blood pressure decrease is caused by NO-related interference on the arteriolar resistance vessels [9,12,13]. Our data support sepsis guidelines, which focus on an early hemodynamic stabilization within the first 3 h [25-27].

The role of the microcirculation as a motor of sepsis is further strengthened by another interesting finding of the present study. Neither l-NIL nor iNOS(−/−) influenced the induction of the pro-inflammatory cytokine IL-6 or the endothelial activation marker ICAM. The reduced levels of the anti-inflammatory cytokine IL-10 under l-NIL might indicate - if at all - an induced inflammatory response. Therefore, it appears that the early inflammatory process itself (cytokine storm, endothelial activation) did not affect the neuronal function directly. Our findings are in line with reports from rheumatoid arthritis patients who present a normal cognitive function during relapses with significantly increased cytokine levels [28,29]. Later on, starting at 24 to 48 h, cytokines are known to trigger delayed apoptotic pathways [30-32].

The finding of a significantly reduced chemokine CXCL-5 expression indicates reduced parenchymal inflammation and, therefore, reduced neuronal stress. CXCL-5 is significantly induced after cerebral ischemia, indicating a hypoxia-triggered inflammation in the brain [33,34]. An alternative explanation might be an anti-inflammatory effect of iNOS blockade due to an inhibition of the NO-related activation of the prostaglandin synthesis [35,36]. However, further research is needed to investigate this issue in more detail.

## Conclusions

We conclude that iNOS blocking has a neurofunctionally stabilizing effect in the early phase of endotoxic shock. Effects are most likely explained by microcirculatory stabilization, strengthening modern sepsis concepts recommending early hemodynamic stabilization of septic patients. Additional anti-inflammatory approaches are warranted to maintain the positive effects and to prevent from other negative effects such as a cytokine-related delayed neuronal apoptosis.
